# Pseudoprogression in CAR-T cell therapy for solid tumor: Case report

**DOI:** 10.3389/fimmu.2024.1504104

**Published:** 2025-01-08

**Authors:** Xuan Zhao, Yanfen Liu, Guohui Qin, Yan Ge, Qinglong Li, Xinfeng Chen, Ximei Tian, Yong Yu, Jiangtao Ren, Yi Zhang

**Affiliations:** ^1^ Biotherapy Center & Cancer Center, The First Affiliated Hospital of Zhengzhou University, Zhengzhou, China; ^2^ State Key Laboratory of Esophageal Cancer Prevention & Treatment, Zhengzhou University, Zhengzhou, China; ^3^ Department of Radiology, The First Affiliated Hospital of Zhengzhou University, Zhengzhou, Henan, China; ^4^ Medical Department, Nanjing Bioheng Biotech Co., Ltd., Nanjing, China; ^5^ Tianjian Laboratory of Advanced Biomedical Sciences, Academy of Medical Sciences, Zhengzhou University, Zhengzhou, Henan, China; ^6^ School of Life Sciences, Zhengzhou University, Zhengzhou, Henan, China; ^7^ School of Public Health, Zhengzhou University, Zhengzhou, Henan, China

**Keywords:** pseudoprogression, CAR-T cell therapy, gastric cancer, evaluation of therapeutic efficacy, cellular immunotherapy

## Abstract

We reported the pseudoprogression in an elderly patient with advanced gastric cancer after chimeric antigen receptor (CAR)-T cell therapy. The hepatic metastases enlarged 1 month after CAR-T cell infusion and then shrunk the next month as seen through computed tomography scanning. Based on a comprehensive evaluation that includes imaging, pathology, serum tumor markers, and clinical symptoms, we arrived at a diagnosis of pseudoprogression after CAR-T cell therapy, which has not been reported in previous studies. In this report, we provide detailed descriptions of the patient’s clinical presentation, imaging findings, treatment process, and follow-up outcomes. We believe that this case holds important implications for CAR-T cell therapy research and offers valuable insights for clinical practice.

## Introduction

Tumor pseudoprogression after immunotherapy in solid tumors is a phenomenon in which tumor lesions are enlarged or newly added to during the course of immunotherapy, followed by tumor shrinkage (1). Pseudoprogression is more common after the use of immune checkpoint inhibitors but has not been reported in chimeric antigen receptor (CAR)-T cell therapy of solid tumors (2, 3).

**Figure 1:**
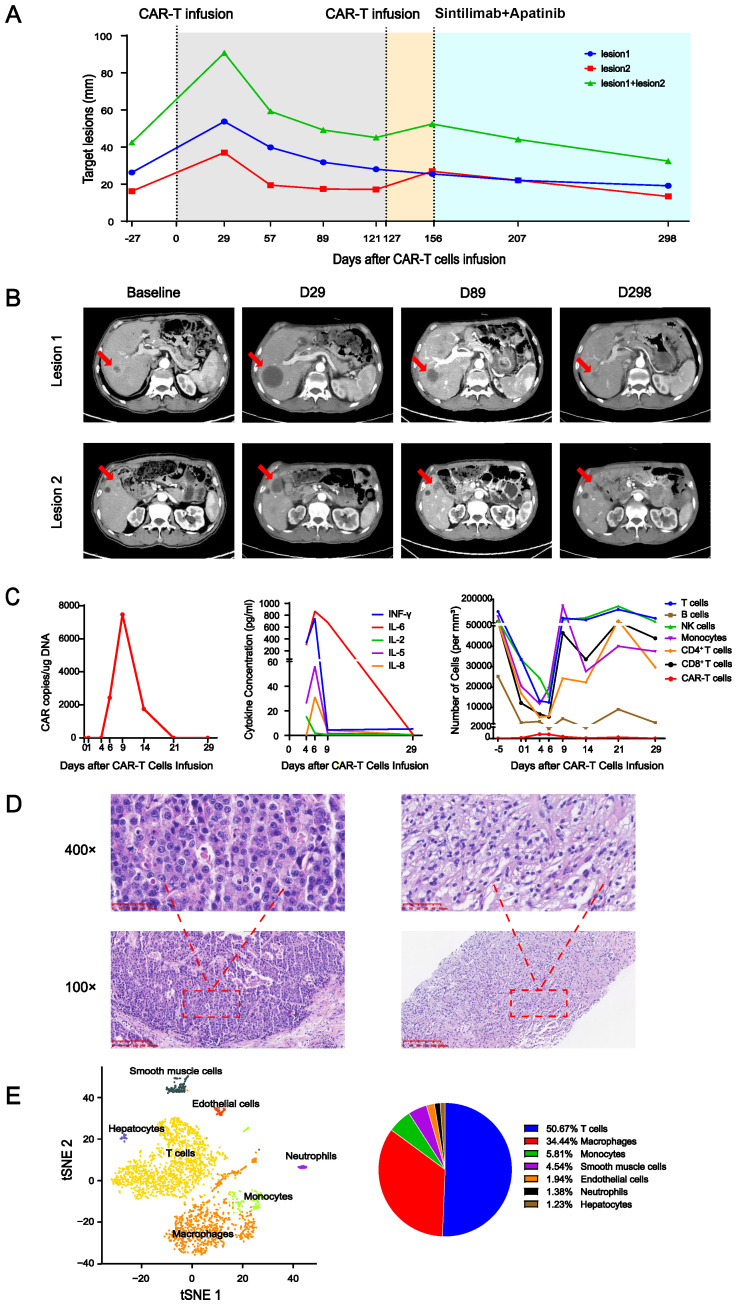
**(A)** Changes in the target lesion on enhanced CT. The gray area indicates the period after the first CAR-T cell infusion, yellow indicates the period after the second CAR-T cell infusion, and blue indicates the period of sintilimab+apatinib treatment. **(B)** Several enhanced CT images of selected key points. Red arrow indicates lesions. **(C)** Peripheral blood parameters. Left panel: CAR copy number over time. Middle panel: dynamics of cytokine levels over time. Right panel: immune cell counts over time. **(D)** Hematoxylin and eosin (H&E) staining of the surgical specimen of gastric cancer (left) was magnified at 100× and 400×. H&E staining of the liver metastasis specimen taken 100 days after CAR-T cell infusion (right) was magnified at 100× and 400×. **(E)** The single-cell sequencing (left) of the liver metastasis specimen taken 100 days after CAR-T cell infusion and its statistical pie chart (right).

An elderly patient was diagnosed with advanced gastric cancer and had undergone radical gastrectomy after neoadjuvant chemotherapy, followed by adjuvant chemotherapy. Liver metastases were found 3 months later; therefore, the patient received sequential treatment with chemotherapy and a tyrosine kinase inhibitor. The tumor continued to progress. Subsequently, the patient was enrolled in a clinical trial of Claudin 18.2 CAR-T cell treated with solid tumor. This clinical trial was approved by the Clinical Research Ethics Committee of the First Affiliated Hospital of Zhengzhou University. The intensity (proportion) of Claudin 18.2 expression in the gastric cancer tissue was 3+ (70%), 2+ (20%), and 1+ (5%). Blood mononuclear cells of the patient were collected on day −25 (25 days before CAR-T cell infusion). After preparatory lymphodepletion with cyclophosphamide, fludarabine, and nab-paclitaxel, an infusion of a total of 8.55×10^8^ (1.5×10^7^/kg) Claudin 18.2 CAR-T cells was administered on day 0. Intermittent fever appeared and peaked on day 3. Grade I cytokine release syndrome (CRS) was assessed and tocilizumab was administered. The levels of INF-γ, IL-5, IL-6, and IL-8 in the peripheral blood peaked on day 6 (**Figure 1C**, middle), the patient’s temperature settled within a week, and the cytokine concentration was also decreased. CAR-T cell expansion in the peripheral blood peaked 9 days after infusion (**Figure 1C**, left) and other cell subsets were relatively low at 0–6 days (**Figure 1C**, right). The patient intermittently took an oral chemotherapy drug on the patient’s own initiative from apheresis to 14 days after infusion.

On day 28, enhanced computed tomography (CT) scanning showed an increase in the total diameter of the two liver metastases from 42.47 mm at baseline to 90.73 mm (**Figures 1A, B**), which was assessed as disease progression according to Response Evaluation Criteria in Solid Tumors (RECIST) 1.1. The patient’s Karnofsky Performance Status score gradually improved, multiple tumor markers decreased in the peripheral blood, and no deterioration of liver function was observed. CT reexamination showed that the total diameters of the two target lesions were 59.27 mm on day 56 and 49.15 mm on day 89 after infusion. On day 100, pathological biopsy of liver metastasis showed a large amount of immune cell infiltration, but no tumor cells compared to the primary tumor (**Figure 1D**), and single-cell sequencing showed a mass of immune cell infiltration dominated by T lymphocytes (**Figure 1E**). This was analogous to the pseudoprogression observed in immune checkpoint inhibitor therapy (1). The total diameter of the target lesions on day 121 was smaller than before, but a new lesion appeared. At this time, no CAR-T cells were detected in the peripheral blood; therefore, after preparatory lymphodepletion, a total of 1×10^9^ (2×10^7^/kg) Claudin 18.2 CAR-T cells were infused on day 127. No any-grade CRS or immune effector cell-associated neurotoxicity syndrome (ICANS) occurred. The CAR-T cell copy number in peripheral blood peaked at day 14 after reinfusion at 4,858.59 copies/μg DNA. On day 156, after reinfusion for 29 days, the target lesions were enlarged again with no CAR-T cells detected in peripheral blood, at which time the treatment regimen of sintilimab (an anti-PD-1 antibody) and apatinib (a tyrosine kinase inhibitors) was started. The therapy was continued for seven cycles, and the tumor continued to shrink at the last follow-up CT imaging on day 298. At the most recent long-term survival follow-up, the patient remained alive at 619 days after CAR-T cell reinfusion.

In this case, the total diameter of the target lesions increased by more than double rapidly after the infusion of CAR-T cell. According to RECIST, it would be assessed as disease progression (4). However, it allows for more time to observe and confirm whether it is true disease progression or potentially an immune-related event according to iRECIST (5). Subsequent observations revealed that the total diameters of the tumor target lesions gradually decreased. Combined with the patient’s clinical symptoms, tumor markers in serum, and pathology, we believed this to be pseudoprogression (6). Interestingly, the lesion of the patient temporarily enlarged again after reinfusion of the CAR-T cell. This case indicates that iRECIST may be more suitable for evaluating the efficacy of CAR-T cell therapy in solid tumors than RECIST (7, 8). After CAR-T cell infusion combined with anti-PD-1 antibody treatment, the application of this combined immunotherapy resulted in sustained disease remission, indicating the feasibility of combined immunotherapy (9, 10).

In conclusion, this case describes the phenomenon of pseudoprogression after CAR-T cell therapy in solid tumors for the first time. This case excluded the possibility of hyperprogression after treatment through biopsy and emphasizes the importance of using iRECIST in evaluating the disease status during CAR-T cell therapy for solid tumors. Additionally, this case demonstrates the feasibility and potential benefits of CAR-T cell infusion combined with anti-PD-1 antibody for the treatment of solid tumors.

## Data Availability

The original contributions presented in the study are included in the article/supplementary material. Further inquiries can be directed to the corresponding authors.
